# Metamorphopsia and Morphological Changes in the Macula after Scleral Buckling Surgery for Macula-Off Rhegmatogenous Retinal Detachment

**DOI:** 10.1155/2021/5525049

**Published:** 2021-06-25

**Authors:** Sisi Xu, Ling Wang, Kangjie Kong, Gang Li, Yingqin Ni

**Affiliations:** ^1^Department of Ophthalmology, Eye and ENT Hospital, Fudan University, Shanghai, China; ^2^Department of Ophthalmology, The First Affiliated Hospital of Wenzhou Medical University, Wenzhou, Zhejiang, China; ^3^Shanghai Key Laboratory of Visual Impairment and Restoration, Shanghai, China; ^4^Key NHC Key Laboratory of Myopia (Fudan University), Laboratory of Myopia, Chinese Academy of Medical Sciences, Shanghai, China; ^5^Research Center, Eye and ENT Hospital, Fudan University, Shanghai, China

## Abstract

**Purpose:**

To observe the changes in metamorphopsia after scleral buckling (SB) surgery for macula-off rhegmatogenous retinal detachment (RRD) and its association with morphological changes in the macula.

**Methods:**

This prospective study included 20 eyes of 20 patients. Before surgery and 1, 3, 6, and 12 months after surgery, metamorphopsia measured by *M*-charts and best-corrected visual acuity (BCVA) and macular microstructures assessed using optical coherence tomography were recorded.

**Results:**

Both the vertical and horizontal *M*-scores improved significantly after SB surgery. BCVA also improved gradually. The mean *M*-score in the eyes with a continuous external limiting membrane (ELM) was smaller than that in the eyes with a disrupted ELM (*P*=0.008). Preoperative and postoperative BCVA did not correlate with the mean *M*-score at any time point. The other studied parameters, namely, the duration of RRD, the height of retinal detachment, central foveal thickness, inner nuclear layer thickness, and continuation of the ellipsoid zone, were also not relevant.

**Conclusions:**

The continuation of the ELM may be a critical factor in determining the severity of metamorphopsia after SB surgery for macula-off RRD.

## 1. Introduction

As the most frequent form of retinal detachment, rhegmatogenous retinal detachment (RRD) is an emergency among retinal diseases [1]. Due to improvements in surgical instruments and methods, the anatomical reattachment rate is high, and patients' visual acuity postoperatively can be restored [2, 3]. Some alternative techniques, such as microscope-assisted ab externo surgery and vitrectomy under air, could further reduce postoperative complications [4, 5]. However, metamorphopsia is a prevalent and persistent symptom identified by patients, especially those with macula-off RRD [6, 7]. The reported incidence of metamorphopsia ranges from 39% to 88%, and it greatly impairs patients' quality of vision [8–10].

Despite being used extensively in clinics, the Amsler grid test can only assess metamorphopsia qualitatively but not quantitatively. Recently, researchers have developed innovative tools to evaluate the degree of deformation, such as MacuFlow, preferential hyperacuity perimetry, and D-charts [11–13]. First introduced in 1999, *M*-charts serve as a handy tool to assess metamorphopsia both quantitatively and sensitively [14]. It has been adopted in studies on metamorphopsia involving the epiretinal membrane (ERM), macular holes, and branch retinal vein occlusion (BRVO) [15–17].

The precise pathophysiological mechanism of metamorphopsia is not clear [18]. Although there is emerging research on the factors associated with metamorphopsia in RRD, most of the results have been from patients who underwent pars plana vitrectomy (PPV) [8, 19]. Sophisticated operations, macular surgery to remove the internal limiting membrane, and various tamponades indicate that many diverse factors can influence metamorphopsia recovery after PPV. Scleral buckling (SB) surgery, which is also an effective method for treating RRD, disrupts the homeostasis of the intraocular environment relatively less [20]. It therefore offers an ideal model for studying the factors that influence metamorphopsia after retinal detachment. The purpose of this study was to observe the changes in metamorphopsia after SB for macula-off RRD and its association with morphological changes in the macula.

## 2. Materials and Methods

### 2.1. Patients

This was a prospective study. Twenty patients (20 eyes) diagnosed with RRD caused by atrophic holes at the Eye and ENT Hospital of Fudan University between March 2018 and May 2020 were enrolled. The study followed the tenets of the Declaration of Helsinki and was approved by the Ethics Review Board of the Eye and ENT Hospital of Fudan University. Signed informed consent forms were obtained from all the patients or their legal guardians.

The inclusion criteria were as follows: (1) a definite diagnosis of macula-off RRD; (2) proliferative vitreoretinopathy Grade B or below; (3) successful retinal reattachment through single SB surgery without serious complications, such as macular displacement, macular oedema, and macular folds. Patients with longstanding RRD, glaucoma, uveitis, macular hole, and other retinal diseases were excluded. Patients' age, gender, affected eye, duration of symptoms, and surgical approaches were collected. Before surgery and 1, 3, 6, and 12 months after surgery, metamorphopsia measured by *M*-charts and best-corrected visual acuity (BCVA) and macular microstructures assessed using optical coherence tomography (OCT) were recorded.

### 2.2. SB Surgery

All the surgeries were performed by two experienced surgeons (Y. Ni and L. Wang) under retrobulbar or general anesthesia conditions. After conjunctival peritomy, the rectus muscles were isolated and looped off. A combination of cryotherapy under indirect ophthalmoscopy and exoplant (segmental silicone sponges or circumferential silicone bands) was used to treat retinal holes. Decisions regarding the external drainage of subretinal fluid (SRF) depended on the surgeons' judgment as to whether the amount of SRF would affect the reattachment of the retina. No intraocular tamponades were used.

### 2.3. *M*-Charts

The *M*-charts (Inami Co, Tokyo, Japan) comprised 19 dotted straight lines with dot intervals at a visual angle of 0.2° to 2.0°. At a distance of 30 cm with the best refractive correction, each patient was asked whether the dotted line was straight or not. If the answer was yes, the examination ended with the visual angles being his/her final score. If the answer was no, subsequent pages were shown to him/her until he/she recognized it as straight. The measurements were taken both vertically (*M*-score vertically, MV) and horizontally (*M*-score horizontally, MH). The examinations were repeated three times. The MV and MH were recorded, and the mean *M*-score (MM) was calculated (mean *M*-score = MV/2 + MH/2). When analyzing the correlations, BCVA and the duration of RRD were compared with the MM. The HRD, CFT, and INL thickness were compared with the MV and the MH separately.

### 2.4. BCVA

Measured with Snellen charts, the BCVA values were converted to the logarithm of the minimum angle of resolution (logMAR) for statistical analysis. Finger counting was converted to 1.98 logMAR [21].

### 2.5. OCT

**Figure 1:**
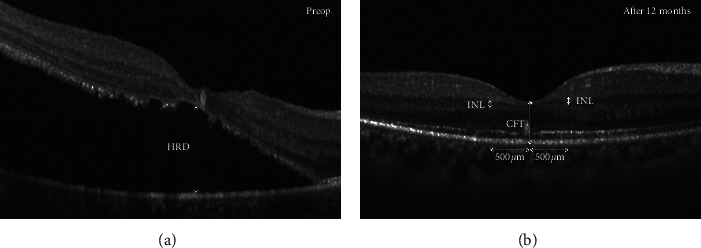
This schema presents the measurement of the parameters on OCT. HRD, height of retinal detachment; CFT, central foveal thickness; INL, inner nuclear layer.

Retinal images were recorded using spectral-domain OCT (Heidelberg Engineering, Heidelberg, Germany). Line scans centered on the fovea in the vertical and horizontal directions were obtained as well as 3D scan. The height of retinal detachment (HRD), central foveal thickness (CFT), and inner nuclear layer (INL) thickness 500 *μ*m from the fovea were manually measured on both the horizontal and vertical scan lines using Adobe Photoshop CS6 software (Adobe Systems, Inc., San Jose, CA).  Figure 1 indicates the measurement of the parameters on OCT. The statuses of the external limiting membrane (ELM) and ellipsoid zone (EZ) within a 3 mm area centered at the presumed fovea were evaluated in both the horizontal and vertical scans. Disruption was defined as the loss or irregularity of each hyperreflective line in at least one scan. All the OCT measurements and judgments were carried out by two investigators (G. L. and L. W.) who were blinded to the clinical data.

### 2.6. Statistical Analysis

The Friedman ANOVA test with Bonferroni post hoc analysis was used to evaluate the pre- and postoperative changes in BCVA and *M*-scores. The relationships between the *M*-scores and BCVA, HRD, CFT, and INL were examined using the Pearson correlation test. The relationship between the *M*-scores and the duration of RRD was examined by the Spearman correlation test. Mann-Whitney *U* test was used to evaluate the average *M*-scores between the two groups based on the drainage of SRF, the extent of SRF retention, and the statuses of the ELM and EZ. The statistical analyses were performed using SPSS software (version 23.0; SPSS Inc, Chicago, IL). *P* < 0.05 was considered statistically significant.

## 3. Results

### 3.1. Patient Demographics

Twenty eyes (14 right eyes, 6 left eyes) of 20 patients (13 males, 7 females) were included in this study. The average age at surgery was 27.6 ± 8.9 years (range 15–47 years). The median duration of blurred vision was 30.0 days (range 7–120 days). All the eyes were phakic preoperatively and postoperatively. Eight cases (40.0%) underwent SRF drainage. Eight cases (40.0%) underwent cryotherapy with segmental silicone bands while the balance (60.0%) underwent cryotherapy with circumferential silicone bands. No serious complications were observed intraoperatively or postoperatively. The demographic and clinical characteristics of the patients are summarized in Table 1.

### 3.2. Changes in BCVA and Metamorphopsia over Time and Their Relationship

**Figure 2:**
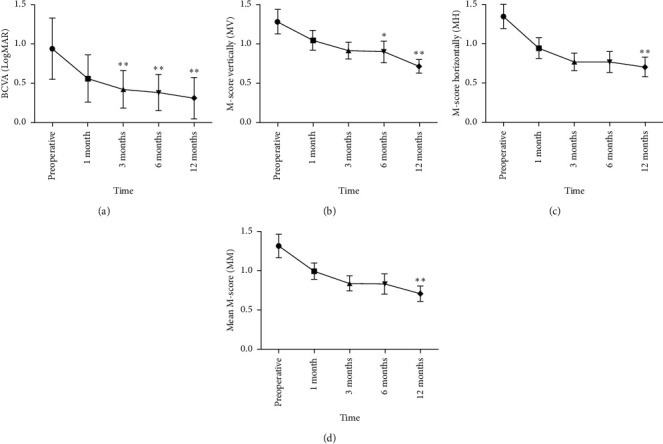
The changes in BCVA (a), the *M*-score vertically (b), the *M*-score horizontally (c), and the mean *M*-score (d) after scleral buckling surgery in eyes with macular-off rhegmatogenous retinal detachment. The Friedman ANOVA test with Bonferroni post hoc analysis was used for the comparison. ^*∗*^*P* < 0.05, ^*∗∗*^*P* < 0.01.

The changes in BCVA and metamorphopsia are shown in Figure 2. BCVA improved gradually from 0.94 ± 0.39 preoperatively to 0.56 ± 0.30 (*P*=0.188), 0.42 ± 0.24 (*P* < 0.01), 0.38 ± 0.23 (*P* < 0.01), and 0.31 ± 0.26 (*P* < 0.01) at 1, 3, 6, and 12 months postoperatively, respectively (Figure 2(a)). The MV and MH were 1.29 ± 0.70 and 1.39 ± 0.69 preoperatively, 1.05 ± 0.56 and 0.94 ± 0.60 at 1 month, 0.92 ± 0.48 and 0.77 ± 0.49 at 3 months, 0.91 ± 0.62 and 0.77 ± 0.60 at 6 months, and 0.72 ± 0.39 and 0.70 ± 0.56 at 12 months postoperatively, respectively (Figures 2(b) and 2(c)). Compared with those before surgery, there was a substantial improvement in MM (Figure 2(d)).

The preoperative BCVA did not correlate with the preoperative MM (*P*=0.401). At 1-month after operation, BCVA had no significant correlation with MM (*P*=0.058). At 3 months, BCVA had no significant correlation with MM (*P*=0.260). Additionally, the relationship between BCVA and MM at 6 and 12 months was of no significance (*P*=0.485 and *P*=0.610, respectively).

### 3.3. Relationship between Metamorphopsia and HRD, Duration of RRD, SRF Drainage, and SRF

The HRD measurements of 7 patients for whom the detached macula was invisible inside the OCT frame preoperatively were excluded. The mean HRD preoperatively of the remaining 13 patients was 485.5 *µ*m (range 127–1016 *µ*m) on the horizontal scan lines and 426.0 *µ*m (range 139–953 *µ*m) on the vertical scan lines. The HRD was not correlated with metamorphopsia in any direction (Table 2).

The duration of RRD did not correlate with the mean *M*-scores at any time point (preoperative, *P*=0.703; 1 month, *P*=0.411; 3 months, *P*=0.077; 6 months, *P*=0.107; 12 months, *P*=0.668). The median time of retinal detachment of 8 patients with SRF drainage was 22.5 days (range 7–120 days). The median time of retinal detachment of 12 patients without SRF drainage was 45.0 days (range 14–120 days). The difference was of no significance (*P*=0.208). At 12 months, the MM were 0.67 ± 0.24 and 0.73 ± 0.54, respectively. The difference was not statistically significant (*P*=0.792).

The patients were divided into two groups, Group SRF- and Group SRF+, based on the presence of SRF. At 1 month and 3 months postoperatively, the difference between the two groups was not significant (Table 3). At 6 months, the SRF had been absorbed in 4 patients but persisted in the remaining 16 patients. The difference was again of no significance (Table 3).

### 3.4. Relationship between Metamorphopsia and Macular Microstructures

**Figure 3:**
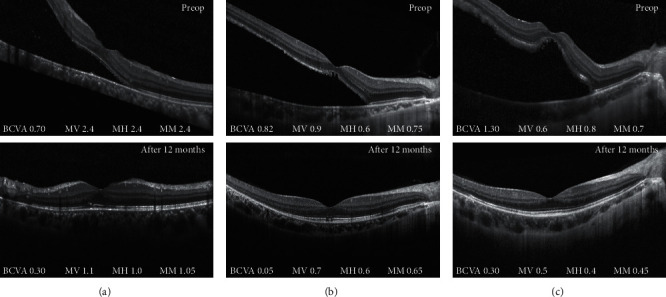
Three representative patients. (a) Image of the right eye of a 28-year-old man. Both the ELM and EZ were disrupted. The mean *M*-score at 12 months was 1.05. (b) Image of the right eye of a 15-year-old man. The EZ was disrupted, and the ELM was continuous. The mean *M*-score at 12 months was 0.65. (c) Image of the right eye of a 19-year-old man. Both the ELM and EZ were continuous. The mean *M*-score at 12 months was 0.45. MV, *M*-score vertically. MH, *M*-score horizontally. MM, mean *M*-score. ELM, external limiting membrane. EZ, ellipsoid zone.

At the 12-month follow-up, the SRF had been completely absorbed in 18 patients but persisted in 2 patients. Of the 2 patients, one was macula-involved and the other was macula-free. The mean CFT of these 18 patients was 207.6 *µ*m (range 143–271 *µ*m) on the horizontal scan lines and 215.1 *µ*m (range 161–286 *µ*m) on the vertical scan lines. The mean INL thickness was 20.8 *µ*m (range 10–39 *µ*m) horizontally and 25.7 *µ*m (range 16–36 *µ*m) vertically. Neither the CFT nor INL thickness correlated with metamorphopsia in any direction (Table 2). Among the 18 patients, 9 eyes had continuous ELM (Group ELM+), and 9 eyes had disrupted ELM (Group ELM−). Furthermore, 8 eyes had continuous EZ (Group EZ+), and 10 eyes had disrupted EZ (Group EZ−). The average MM were 0.49 and 0.99 for Group ELM+ and Group ELM−, respectively. The difference was statistically significant (*P*=0.008). Average MM were 0.55 and 0.90 for Group EZ+ and Group EZ−, respectively, and the difference was of no significance (*P*=0.083) (Table 3). Three representative patients are presented in Figure 3.

## 4. Discussion

In the present study, metamorphopsia was gradually alleviated after SB surgery in the patients with macula-off RRD but rarely disappeared in the 12-month follow-up. BCVA was not correlated with metamorphopsia at any time point. The other parameters, namely, duration of RRD, HRD, SRF, CFT, and INL thickness, were also not correlated with metamorphopsia. The severity of metamorphopsia was associated with the integrity of the ELM.

Generally, the *M*-score in our study was slightly higher than that reported by other researchers with respect to RRD patients but comparable to that reported by Lina et al. [10]. The different subjects included in the studies may explain this difference. In the present study, only macula-off RRD patients were included. Some studies included both macula-off and macula-on RRD patients [8, 9]. Research has shown that macula-off patients are more prone to experiencing metamorphopsia [8, 9]. Moreover, this study focused solely on patients undergoing SB surgery. Compared with direct SRF drainage during PPV, it took more time for the SRF to be absorbed with SB surgery [22, 23]. Aside from the above, another reason may be that M-charts are more sensitive in detecting metamorphopsia than the Amsler grid [24].

Whether there is a correlation between BCVA and metamorphopsia remains an open question. In their study, Okuda et al. found no statistical correlation between BCVA and the *M*-scores in eyes with macula-off or macula-on RRD at 6 months and 12 months postoperatively [9]. Similar results have also been reported in patients with macular hole and diabetic macular edema [16, 25]. However, Murakami et al. found a significant relationship between the *M*-score at 12 months postoperatively and preoperative BCVA but not BCVA at 12 months [26]. In our study, BCVA did not correlate with the *M*-score at any time point. The only *P* value that seemed to show a statistical correlation was found at 1 month after operation. This suggested that there may be a specific relationship between these two indexes of visual function. After all, improvements in visual acuity and relief from metamorphopsia both depended on the recovery of the retinal structures, but the sense of deformation seemed to be more subjective, and *M*-score varied obviously among individuals. Alleviation of metamorphopsia also required more refined microstructural restoration. All these factors made it difficult to find a significant correlation between BCVA and *M*-scores.

In our study, the interval from blurred vision to surgery was 7–120 days, which was comparable to other studies [7, 27]. Our results showed that the duration of RRD did not correlate with *M*-scores at any time point. At present, few studies have been published concerning the correlation between the duration of detachment and the severity of metamorphopsia in RRD [27]. Some studies reported that metamorphopsia correlated significantly with duration of symptoms in macular hole, central serous chorioretinopathy, and BRVO [17, 28, 29]. However, So et al. found no correlation in ERM [30]. We have consulted the literature when writing this paper. However, we have not found a particularly good theoretical basis to explain this phenomenon. A larger sample size study would be needed to fully explain this.

Since no study has analyzed the association between *M*-scores and HRD, it was difficult to compare the results of our study with those of others. Only a few studies in the literature mention SRF when studying metamorphopsia. Zhou et al. suggested that the presence of SRF is one of the independent predictors of metamorphopsia after RRD surgery [27], but they only used the Amsler grid to assess metamorphopsia qualitatively in their study, and no quantification of metamorphopsia is therefore available. Amir et al. investigated whether SRF drainage during RRD repair surgery would influence postoperative metamorphopsia [31]. They found no association between SRF drainage and the *M*-scores. In our study, whether SRF was absorbed completely had no effect on *M*-scores. The exact effect of SRF on metamorphopsia remains controversial and needs further elucidation.

Recent studies have shown that the postoperative metamorphopsia of RRD is mainly related to the structures of the outer retina such as the ELM, EZ, and interdigitation zone [9, 26]. Because of the differing designs of the studies, the conclusions are not completely consistent. In our study, the continuation of the ELM was a critical factor in determining *M*-scores, and the continuation of the EZ seemed to relieve metamorphopsia. However, the small sample size could not yield a statistical difference. It has been speculated that metamorphopsia may result from the displacement of photoreceptors and the false localization of the image seen by these displaced photoreceptors. This may account for the importance of ELM and EZ in metamorphopsia. In eyes with an ERM, CFT and INL thickness have been found to be good indicators of metamorphopsia [15, 32]. However, we found no association between CFT and INL thickness and *M*-scores in our patients with RRD. The different pathogenic causes of ERM and RRD may be the main reason for this. The exact mechanism of metamorphopsia is complex and unexplained. Future studies using more advanced imaging devices and focusing on individual neurons are needed to reveal this.

We acknowledge several limitations in this study. First, our exacting standard of inclusion accounted for the relatively small number of patients. Patients with SB surgery indications but with longstanding retinal detachment, those with intraoperative or postoperative complications, and those who were unable to fulfill the 12-month follow-up were all excluded. Our follow-up was also not sufficiently long to observe metamorphopsia disappear completely since metamorphopsia may exist for a long time [6]. In addition, our OCT parameters were measured manually. Further studies with larger samples, longer postoperative follow-ups, and the automatic measurement of OCT are clearly warranted.

In conclusion, the continuation of the ELM may be a critical factor in determining the severity of metamorphopsia after SB surgery for macula-off RRD.

## Figures and Tables

**Table 1:** Demographic and clinical characteristics of the patients.

Characteristics	Value
Number (patients/eyes)	20/20
Age (years)	27.6 ± 8.9
Duration of blurred vision (days)	Median 30.0
Sex (male/female)	13/7
Affected eyes (right/left)	14/6
Lens status (phakic/pseudophakic)	20/0
Subretinal fluid drainage (yes/no)	8/12
Type of surgery (segmental or circumferential)	8/12

**Table 2:** The relationship between metamorphopsia and the OCT parameters.

	MV before operation		MH before operation		MV at 12 months		MH at 12 months	
	*R*	*P*	*R*	*P*	*R*	*P*	*R*	*P*
*HRD*								
Vertical	0.004	0.989	−0.072	0.815	—	—	—	—
Horizontal	−0.177	0.562	−0.135	0.660	—	—	—	—

*CFT*								
Vertical	—	—	—	—	−0.341	0.196	−0.297	0.264
Horizontal	—	—	—	—	−0.386	0.113	−0.101	0.691

*INL thickness*								
Vertical	—	—	—	—	−0.163	0.545	−0.212	0.399
Horizontal	—	—	—	—	−0.426	0.100	−0.153	0.544

HRD, height of retinal detachment; CFT, central foveal thickness; INL, inner nuclear layer; MV, *M*-score vertically; MH, *M*-score horizontally.

**Table 3:** The relationship between metamorphopsia and the macular microstructures on OCT.

Factors	Number	Mean *M*-score	*P*
*SRF at 1 month*			
－	2	0.93	0.853
＋	18	1.00	

*SRF at 3 months*			
－	3	0.98	0.305
＋	17	0.81	

*SRF at 6 months*			
－	4	0.93	0.249
＋	16	0.81	

*ELM at 12 months*			
Continuation	9	0.49	0.008^*∗∗*^
Rupture	9	0.99	

*EZ at 12 months*			
Continuation	8	0.55	0.083
Rupture	10	0.90	

SRF, subretinal fluid; ELM, external limiting membrane; EZ, ellipsoid zone. ^*∗*^*P* < 0.05, ^*∗∗*^*P* < 0.01.

## Data Availability

The data used to support the findings of this study are available from the corresponding author upon request.
